# ‘Practical’ resources to support patient and family engagement in healthcare decisions: a scoping review

**DOI:** 10.1186/1472-6963-14-175

**Published:** 2014-04-15

**Authors:** Katharina Kovacs Burns, Mandy Bellows, Carol Eigenseher, Jennifer Gallivan

**Affiliations:** 1 Health Sciences Council and Interdisciplinary Health Research Academy, 3-389 Edmonton Clinic Health Academy, University of Alberta, 11405 – 87 Avenue, Edmonton T6G 1C9, Alberta, Canada; 2 Patient Engagement Department, Alberta Health Services, Edmonton, Alberta, Canada

**Keywords:** Patient engagement, Scoping literature review, Resource Kit, Engagement tools, Education, Infrastructure, Evaluation

## Abstract

**Background:**

Extensive literature exists on public involvement or engagement, but what actual tools or guides exist that are practical, tested and easy to use specifically for initiating and implementing patient and family engagement, is uncertain. No comprehensive review and synthesis of general international published or grey literature on this specific topic was found. A systematic scoping review of published and grey literature is, therefore, appropriate for searching through the vast general engagement literature to identify ‘patient/family engagement’ tools and guides applicable in health organization decision-making, such as within Alberta Health Services in Alberta, Canada. This latter organization requested this search and review to inform the contents of a patient engagement resource kit for patients, providers and leaders.

**Methods:**

Search terms related to ‘patient engagement’, tools, guides, education and infrastructure or resources, were applied to published literature databases and grey literature search engines. Grey literature also included United States, Australia and Europe where most known public engagement practices exist, and Canada as the location for this study. Inclusion and exclusion criteria were set, and include: English documents referencing ‘patient engagement’ with specific criteria, and published between 1995 and 2011. For document analysis and synthesis, document analysis worksheets were used by three reviewers for the selected 224 published and 193 grey literature documents. Inter-rater reliability was ensured for the final reviews and syntheses of 76 published and 193 grey documents.

**Results:**

Seven key themes emerged from the literature synthesis analysis, and were identified for patient, provider and/or leader groups. Articles/items within each theme were clustered under main topic areas of ‘tools’, ‘education’ and ‘infrastructure’. The synthesis and findings in the literature include 15 different terms and definitions for ‘patient engagement’, 17 different engagement models, numerous barriers and benefits, and 34 toolkits for various patient engagement and evaluation initiatives.

**Conclusions:**

Patient engagement is very complex. This scoping review for patient/family engagement tools and guides is a good start for a resource inventory and can guide the content development of a patient engagement resource kit to be used by patients/families, healthcare providers and administrators.

## Background

Patient-centred care implies that patients and their families are the focus of the health care system as recipients of its services, programs and delivery approaches [1]. The aim is to ensure that service delivery and decisions are made around the principles of patient and family-centred healthcare, and focus on safety compliance, ‘best practice’ or evidence-based interventions, policies and positive health outcomes [2]. The outcome of this model is that patients and their families are actively and meaningfully engaged in discussions and decisions concerning policies, programs, service delivery and implications of the care provided. The challenge for healthcare institutions is to locate or develop, and implement the mechanisms, tools and resources essential for preparing and supporting patients, their families, healthcare providers and healthcare administrators to effectively and successfully practice patient engagement. This challenge became the ‘Call to Action’ by the Canadian Health Services Research Foundation to health authorities and institutions between 2009 and 2013.

One institution which took up this challenge was Alberta Health Services (AHS), the provincial health organization in Alberta, Canada. AHS identified patient engagement as one of its core principles aligned with quality and safety of health service delivery, and created a Patient Engagement Framework for the organization. In this Framework, patient engagement was defined as “a broad two way practice guided by a set of principles, processes and activities that provide an opportunity for stakeholders to be involved in meaningful interactions. Engagement considers and incorporates the values and needs of patients, clinicians, and communities into health services decision making to enhance transparency and accountability” [3]. In line with this definition, AHS defined ‘patient’ in the broadest sense, as “all individuals including clients, residents and members of the public who receive or have requested healthcare or services from AHS and its health care providers”. In 2009 AHS established the Patient Engagement Department to help advance patient engagement throughout the organization. As part of this work, Patient Engagement staff identified the need for a Resource Kit for patients, providers (e.g. clinicians), staff and leaders (e.g. administrators) to guide them in how to initiate and implement patient engagement within AHS.

Questions were posed regarding the development of a resource kit that would provide the basics as well as some advanced knowledge, skills, tools and resources needed for engaging patients/families. If health care organizations, and more specifically health care providers and administrators, want to involve patients and families in providing advice or input on improving care delivery or access, what are some effective approaches to do this? What preparation is needed to effectively engage patients and families in AHS decisions? Are there existing models, tools and guides which can be used or adapted?

These questions were incentives for conducting a systematic scoping literature review, of both published and grey literature, as described by Arksey and O’Malley [4]. The intent of this scoping review was to identify what tools and guides, education and infrastructure resources existed to engage patients and families in healthcare delivery and other decision-making processes. The ultimate goal was to have an inventory of existing materials which would inform the contents for a patient engagement resource kit to be used by patients, providers and leaders in their efforts to successfully implement and evaluate patient engagement initiatives across AHS. This paper describes the systematic scoping literature review and synthesis of the information into relevant thematic clusters which can be considered for incorporation into a patient engagement resource kit. The actual resource kit development and its pilot or evaluation to determine the quality of the tools and guides for patient engagement is an ongoing process within AHS, and is, therefore, not part of this review or paper.

## Methods

The scoping review was selected as the most suitable method for this study, because by definition it is used “to map *rapidly* the key concepts underpinning a research area and the main sources and types of evidence available, and can be undertaken as stand-alone projects in their own right, especially where an area is complex or has not been reviewed comprehensively before” [5]. The refined approach of Arksey and O’Malley [4] was applied, which includes: (1) identifying the research question/s, which is/are generally broad in nature; (2) identifying topic-relevant studies through a search which is as comprehensive as possible; (3) selecting and rejecting studies using a set of inclusion/exclusion criteria, based on familiarity with the literature; (4) reviewing the sorted and sifted data through charting based on key issues and themes; and (5) analyzing the results through thematic analysis, and reporting findings descriptively and numerically. As a final step, a consultation exercise is recommended involving key stakeholders to inform and validate study findings. Based on the study research questions posed, which was the first step in the scoping review, the overall intent of this study was to provide a comprehensive exploration of the diverse published and grey literature to select the patient engagement tools, education and infrastructure resources that would become the content of a resource kit that would be useful to patients/families, providers and leaders within AHS in their efforts to implement and evaluate patient engagement. The actual resource kit development and its pilot or evaluation to determine the quality of the tools and guides for patient engagement is not part of this review or paper, and therefore, the final step of consultation in the scoping review process is part of the ongoing process within AHS, and reported separately. The following outlines the process taken with the various steps for a scoping review:

### Searching and selecting the published and unpublished (Grey) literature

Searching the published and grey literature required the expertise of a health research librarian. Before the application of search terms, parameters for the published and unpublished grey literature databases or search engines were established through the specific inclusion criteria which limited the literature search to the dates selected and English language preference.

**Figure 1:**
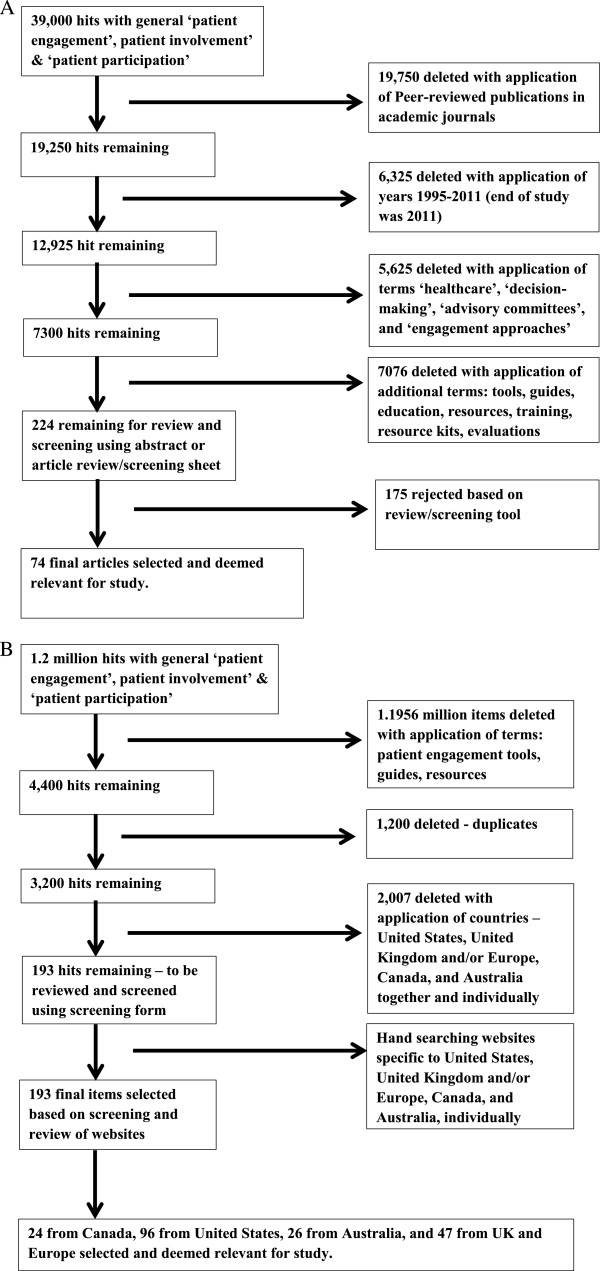
**Flowcharts of published and grey literature screening and selection. A**. Flowchart of the published literature screening and selection process. Application of search terms and inclusion/exclusion criteria as well as review worksheet analysis, resulted in 74 selected articles. **B**. Flowchart of the grey or unpublished literature/material screening and selection process. Application of specific search terms and inclusion/exclusion criteria, as well as review worksheet analysis, resulted in 193 items selected.

The published literature was then searched through Embase, Medline, ERIC, Web of Science and ProQuest using the terms ‘patient engagement’, ‘patient involvement’, and ‘patient participation’. This search was refined through the application of specific search terms such as ‘healthcare’, ‘decision making’, ‘advisory committees’, and other terms pertaining to more specific organizational engagement approaches, education/training, tools, guides and infrastructure supports and evaluation considerations. Some journals known to contain patient engagement studies were hand searched (e.g. *Journal of Participatory Medicine* and *International Journal for Quality in Health Care*). Table 1 contains the search terms applied to the databases. Figure 1(A) depicts the flowchart for the screening process applied to the published literature.

**Table 1:** Search terms and general inclusion/exclusion criteria for published and unpublished (Grey) literature search

**Published literature**	**Grey (Unpublished) literature**
**Search terms used**	**Search terms used**
Applying ‘patient engagement’, ‘patient involvement’, ‘patient participation’ to those below:	Applying ‘patient engagement’, ‘patient involvement’, ‘patient participation’ initially followed with ‘healthcare’, ‘decision making’, advisory committees’, and other terms pertaining to more specific engagement approaches, education/training, tools, resources, and infrastructure supports and evaluation considerations.
• ’knowledge and skills…’	
• ‘design, delivery and/or evaluation processes.	
•‘…readiness for meaningful engagement’.	
• ‘Process and impact of patient engagement’.	Also applied terms to specific countries known for patient/public engagement strategies.
• ‘…resources (e.g. tools), needed by patients, providers, staff and leaders for effective patient engagement/involvement/ participation	Australia
	United Kingdom and rest of Europe
	United States
• ‘…preparation (e.g. education) needed by all stakeholders….’	
• ‘support (e.g. infrastructure) needed by everyone for ….’	
• ‘…in patient-centered system redesign; …to build capacity; …to “pilot” a resource kit; …evaluate the impact’	
• ‘Patient engagement/involvement/ participation resource kit’	
• ‘…best practices…validated tools…proven approaches’	
• ‘Recruit patients…’	
• ‘…spectrum or level of engagement…ready for successful engagement’	
• ‘…knowledge and skills needed	
• ‘…engagement at different levels within the organization (governance, to program-based planning and evaluation)	
• ‘…collaboration and partnering…’	
• ‘…organizational policies and practices…’	
• ‘…resources…’	
• ‘…capacity…’	
**Inclusion criteria for articles**	**Inclusion criteria of literature**
• From 1995-2010	• From 1995-2010
• Written in English	• Written in English
• Abstracts containing one or more of the key search terms or areas as identified in the research proposal.	• Contains one or more of the key engagement-related and healthcare terms or areas.
• Studies that refer to the involvement of patients at the program or governance levels	• From United States, Canada, Australia, and Europe (with special emphasis on United Kingdom).
**Exclusion criteria of articles**	**Exclusion criteria of literature**
• Incorporate public or consumer engagement in areas outside of healthcare.	• Incorporates public or citizen engagement or similar areas rather than patient engagement, and in areas outside of health.
• Refer to involvement of the patients outside of the governance or program level.	• Refers to the involvement of the patients outside of the governance/program level.
• Involve patients in their own treatment and care aspects or in personal healthcare decision making.	

The grey literature was searched using the same and additional search terms (toolkits, resources, education, and supports) applied to Google and MSN search engines. Specific countries were also added to the list of search terms – United States (U.S.), Australia and the United Kingdom (U.K.) and Europe were selected because these countries are well-known in the literature and at conferences for their more advanced patient engagement practices and resources. Canada was selected because it was the country where this study took place, and also because patient engagement was a relatively new approach in Canadian healthcare systems at the time, but had some examples worth noting. Websites were manually searched in addition to the Google and MSN searches. Table 1 also contains the search summary for the grey literature and Figure 1(B) depicts the flowchart for the screening process involved with this type of literature.

### Article review, selection and analysis

All selected published abstracts were read and rated independently by three of the five members of the project literature review working group, thereby ensuring inter-rater reliability through consensus and minimization of bias [6]. Each reviewer rated the abstracts independently using the inclusion/exclusion criteria identified in Table 1, by choosing ‘yes’ include the source, or ‘no’ do not include it, or ‘maybe’ include it. Sources that did not have an abstract were automatically included for review. The ‘majority rule’ was used to decide whether to include or exclude a particular article abstract. All abstracts were discussed by the three reviewers. If there were discrepancies in ratings of an article or item, then the reviewers discussed the source as a group to reach consensus on the rating. To this stage of the screening and selection reduction process, 224 published and 193 grey or unpublished items were selected for more detailed analysis and screening.

In order to chart and begin analysis of the content within the final 224 published articles selected as per the search terms, inclusion and screening criteria, a literature analysis worksheet was designed to gather specific information from each article which other review worksheets would not be able to provide. Rather than examining the methodology, sample sizes or quality of study, as in designed or validated review worksheets or guides, the worksheet designed for this scoping review was intended to gather information on the type of patient engagement (general or specific), type of information (e.g. tools, guides, resources, support, preparation), level within organization (e.g. governance, organization/system, programs), targeted study participants (e.g. patients, staff, leaders), study setting of the article (e.g. region, hospital) and level of engagement (e.g. information sharing, consultation, involvement, active engagement, partnership). Through this process, additional articles were rejected, reaching a final number of 74 to be analysed.

A similar worksheet was used with the 193 grey literature documents selected for review. In addition to topic categories being identified such as patient recruitment, engagement levels, or evaluation, the grey literature analysis worksheet incorporated checklists for identifying specifically whether items reviewed were reports, websites, toolkits, resources, education materials, evaluation and other items for each country selected. All 193 grey literature items were retained for final analysis.

All of the retained articles or items and their review worksheets (74 for published articles and 193 for grey items) were analyzed using content thematic analysis, a well-known qualitative analysis approach [7]. The initial codes from this analysis were derived from the initial patient engagement terms and processes identified for the search and applied to all the articles/items and the worksheets. The themes were identified from the most commonly used codes across all of the articles/items and review worksheets. Specific patient engagement items categorized as tools, education or infrastructure were further identified and classified as inclusive of each theme. Summary tables of these various classified areas were constructed for reporting purposes.

### Limitations or challenges identified

Several limitations or challenges were identified as part of the study process. The first was the language and terminology specific to “patient engagement”. The initial search of the published literature using ‘patient engagement’ resulted in very few hits. More hits were identified with “patient participation” and “patient involvement”. Other terms inclusive of patient engagement were identified as the search continued, and included “consumer participation/ involvement/engagement”, “citizen participation/involvement/engagement”, and “public participation/involvement/engagement”. This meant that the search, if limited to only “patient engagement” would not be comprehensive for this scoping literature review. Hence, all related or similar terms were searched, yielding many more hits for selection. A manuscript on this topic was published by the study team [8].

Another limitation was found with the semantics and overlap of different terms for “resources”, “resource kit”, “tool kit”, “guides” and “resource tool kit”, all related to education/information sources, training packages, models, strategies, approaches or processes, guides, workbooks and other such sources on patient engagement in the broadest sense. Regardless of overlap, all terms needed to be searched for optimal access to all relevant sources for this review.

Another challenge presented with the integration of the published and grey literature rather than keeping these two sets of literature separate for categorizing and clustering of categories into themes. There is no evidence to suggest there is a difference in which approach is used for the identification of the themes destined for use in aligning the contents of the “resource kit for patient engagement”. The themes were selected because they best described the common findings from both published and grey literature.

The larger study focusing on the development and evaluation of a practical patient engagement resource kit for patients/families, providers and leaders received ethics approval through three separate ethics review boards in Alberta, Canada: the Community Research Ethics Board of Alberta (Protocol 3 1015); University of Alberta Health Ethics Review (Pro00018481_CLS6); and Calgary Health Research Ethics Board – Conjoint Health Ethics Board (I.D. # E-23545).

## Results

Figure 1(A) and (B) illustrate the article selection, screening, elimination and final yield for both the published and grey literature. Through the series of screenings when applying the search terms, inclusion and exclusion criteria, and literature review worksheets, 74 published and 193 unpublished articles/items were retained for the final thematic content analysis. Seven themes were identified from this content analysis (*Definition of Patient Engagement, Stakeholder Roles and Expectations, Meaningful and Appropriate Engagement, Models of Engagement, Benefits and Barriers to Patient Engagement, Evaluation of Patient Engagement and Engagement Resourcing*), each summarized into tables (some of which are provided as examples in this paper) which would help inform the key content sections for the proposed Patient Engagement Resource Kit. Within each of the seven themes were sub-themes which were clustered into three main groups more in line with the expected content for a patient engagement Resource Kit – tools (including models, engagement approaches, barriers, benefits, evaluation guides), education (including stakeholder roles and expectations, defining and understanding patient engagement, overcoming barriers or turning them into enablers, using evaluation results to improve process and outcomes), and infrastructure (supports, finances, institutional support, capacity, resources). Through the synthesis of the literature, 15 different terms and definitions for ‘patient engagement’ were found along with 37 different engagement toolkits and frameworks (See Table 2), and 17 models, each unique to target groups, with or without tools or guides, and some with specific process or evaluation frameworks or models (original or adapted).

**Table 2:** Various toolkits for engagement and evaluation of engagement

**A. General toolkits**	**B. Evaluation toolkits**
The Value + Toolkit [9,10]	A Toolkit for Family Involvement in Education [11]
Patient and Public Involvement: Toolkit for Staff [12]	Framework for: Public and Service User Involvement in Health and Social Care Regulation in Ireland [13]
Health Canada Policy Toolkit for public Involvement in Decision Making [14]	Citizen Engagement Progress Measurement Framework [15]
Framework for: Public and Service User Involvement in Health and Social Care Regulation in Ireland [13]	Improvement Leaders’ Guide for Evaluation [16]
Australian Institute of Health Policy Studies Consumer Engagement Framework [17]	Using Patient Feedback: A Practical Guide to Improving Patient Experience – Picker Institute Europe [18]
Public and Patient Engagement Getting it Right: Principles of Engagement [19]	Improvement Leaders’ Guide to Measurement for Improvement [20]
A Staff Guide to Involving Service Users, their Carers and the Public in Cambridgeshire Community Services NHS Trust [21]	
Toolkit for Patient Engagement – NHS [22]	
Community Engagement Handbook for Queensland Health District Health Council members [23]	
Engagement Framework and Toolkit – Capital Health [24]	
Rotherham Community Health Services Handbook for Patient Engagement (Toolkit) [25]	
Improvement Leaders’ Guide Involving patients and carers – general improvement skills – NHS Modernisation Agency [20]	
The Participation Toolkit - Supporting Patient Focus and Public Involvement in NHS Scotland [26]	
Patient and Public Engagement Toolkit for World Class Commissioning (NHS) [27]	
How to Develop a Community Based Patient Advisory Council [28]	

### Definition of patient engagement

Upon reviewing the literature, it was evident that there were a number of different terms and definitions for ‘patient engagement’. This topic was published as a separate manuscript [8]. Although the term ‘patient engagement’ was commonly used in discussions related to patients interacting and being meaningfully involved in health care initiatives, it was rarely used in the literature. Fourteen other terms were identified and defined by 26 different sources on the concepts of patient engagement including Citizen Engagement; Consumer Engagement; Involvement; Meaningful Patient Involvement; Participation; Patient and Public Engagement; Patient and Public Involvement (PPI); Patient-Centred Care; and Patient Involvement. Not only was the terminology for ‘patient engagement’ confusing, so was trying to define it. The terms ‘involvement’, ‘engagement’, and ‘participation’ were often used interchangeably. Forbat et al. [29] concluded that “a range of ways of conceptualizing involvement are used interchangeably in policy and practice without due recognition of the very different meanings of public consultation, patient/carer involvement in treatment decision-making, and patient/carer involvement in service design and development” [29], p. 2547. As well, the terms ‘meaningful’ and ‘involvement’ did not mean the same to all stakeholders. “Meaningful involvement is not considered as an objective in itself in the contexts of projects and therefore have not been carefully planned for, resourced and evaluated” [30], p. 270. The European Patient’s Forum highlighted that “while there is diversity about the manner in which to interpret and implement patient involvement into the healthcare system, there is still a common challenge concerning the concept of meaningful patient involvement” [9], p. 105.

### Stakeholder roles and expectations

A lack of consensus and understanding about terminology, the goals and expectations and roles and responsibilities of stakeholders were perceived as barriers to achieving meaningful and successful patient engagement. Forbat et al. concluded that “one of the greatest barriers to truly integrating patient involvement into health services, policy and research is the conceptual muddle with which involvement is articulated, understood and actioned” [29], p. 2547. For the purposes of informing a resource kit for ‘patient engagement’, consistent terminology was viewed as an important consideration in setting up patient engagement initiatives. Choosing ‘patient engagement’, for example, included all forms of involvement, participation, collaboration and engagement, and ‘patient’ in this context included families or family caregivers as well as others in the public domain. Different role descriptions and terms of reference for specific patient engagement initiatives in the literature articulated how patients and their families would be engaged or were expected to engage, and what the objectives, expectations or outcomes of the initiative were anticipated to be regarding patient engagement [29,30]. Expectations between patients and their families and organizations must be coordinated, hence, the differences in toolkits, frameworks and approaches, as identified in Table 2.

### Meaningful and appropriate engagement

There was a desire to ensure patients and their families provided their perspectives to help design and improve health services; however this was not easy [31]. Broad representation of individuals with a variety of health related experiences would ensure a responsive approach to the needs of service users. Finding the right patient or consumer without an ‘axe to grind’ and who could represent the ordinary patient was the goal [32]. Including one or more patients who had contextual knowledge and experience related to the engagement activity would be of most benefit, for example, “a consumer who has undergone transplant surgery, would be far better able to advise on the needs of consumers in these circumstances, than they would to provide advice on proposed changes to mental health legislation” [32], p. 128. Asking the right questions, such as, who wanted to be involved, and who should be involved, were integral to helping the organization meet its goals for public involvement and accountability [33].

The overview provided by Chafe et al. [34] indicated that the public wanted to be involved in varying degrees, with 25 percent of the public wanting to be involved in healthcare decision making but less than 10 percent wanting to be involved in difficult funding decisions, as they feel ill-equipped to contribute.

Attitudes toward patient engagement were not universal. Patient and provider perspectives of health, treatment, role, and organizational attributes differed and guided personal attitudes towards involvement. Farrell [35] suggested it was the attitudes of those in the care relationship, providers and patients, who played a central role in engagement; while a patient centered approach was one that “makes patients feel that they matter, that professionals are being honest with them and that meaningful discussion is possible”. Staff that reacted to patients and their families in an “impatient, patronizing or disrespectful manner” inhibited future engagement opportunities (p. 23).

Based on feedback from participants of engagement opportunities, their involvement experience had been positive but they “become increasingly impatient when they perceive themselves to be a rubber stamp for decisions that are already taken” [36], p. 14. Abelson and Eyles [36], believed that exposure to the health system through engagement opportunities enabled participants to have a greater understanding of the complexity of health sector decision-making and increased respect for decision makers.

Descriptions of the approach to involving stakeholders in decision making at the National Institute for Health and Clinical Excellence (NICE) stressed that “involving people is a serious business” [37], p. 59, and required open-mindedness as well as willingness to change and accommodate. The Institute for Patient and Family Centred Care [38] suggested that incorporating patients as ‘champions’ for engagement within the health system helped to avoid tokenism; for example, the patient advisor was involved in all stages of a project or initiative (from planning to evaluation).

### Models of engagement

A variety of patient engagement models were used by organizations, health systems and governments in the countries selected for this review, as shown in Table 2. Since Arnstein’s Eight Ladders of participation [39] was first developed to understand and explain citizen involvement, many adaptations have been created to clarify the meaning of involvement. The literature recognized 19 different models of engagement, some models being adaptations of original models. Aside from Arnstein’s Ladder, one of the more popular and adapted models was from the International Association for Public Participation [40] or IAP2 which used ‘inform’, ‘consult’, ‘involve’, ‘collaborate’ and ‘empower’ as levels of involvement. One adaptation of the IAP2 model was found in Health Canada’s *Policy Toolkit for Public Involvement in Decision Making* using communication (inform or educate), listening (gather information), consulting (discuss), engaging and partnering [14]. Using the levels of engagement for IAP2, there were some tested and documented methods of engagement under each level which can inform the Patient Engagement Resource Kit (depicted in Table 3).

**Table 3:** Methods of engagement for each engagement level across the international association for public participation (IAP2) spectrum

**Inform**	**Consult**	**Involve**	**Collaborate**	**Empower**
Mass Media (commercials, advertisements, mailings) [14,41]	Focus group [42]	Forums for debate [13]	Patient advisory councils/committees [12,24,28,37,38,41,43]	Citizen jury [12,14,44]
Website [13]	Patient surveys [12,14,23,26,44,45]	Health panels [12]	Expert patients [12]	Consumer managed project/service [32]
Press releases [13]	Feedback and complaints (i.e. interviews, comment cards etc.) [26,29]	Shadowing patients [12]	Charrette [14]	Citizen’s panels [12,14,44]
Mail outs [13,14]	Story-telling [13,38]	Workshops [14,23,43,44]	Constituent assembly [14]	Consensus conference [14,44]
Fact sheets [1,13,14,26]	Social media (Facebook, Twitter, etc.) [38]	Public meetings [23,26]	Delphi Process [14]	Deliberative polling [14,44,46]
Hotline [14,32]	Planning meetings/ Forums [23,26]		Retreats [14]	Search conference [14]
Displays and exhibitions [38]	Suggestion boxes [26,42]		Round tables [14]	Study circles [14,24]
Presentations [38]	Patient diaries [26]		Impact assessments [41]	Study groups [35,43]
	Mystery shopping [26]		Ethics committees [41]	Sustainable community development [14]
			World café [24,26]	Think tanks [14]
			Town hall meetings [24]	
			Revolving conversation [24]	

### Benefits and barriers to patient engagement

Patient engagement was generally considered beneficial to the health care system in its policy and planning activities, but barriers were also identified. It was because of the known benefits and the management or resolution of barriers that specific enablers of patient engagement could also be identified. A 2010 Cochrane Review of patient involvement found that there were potential benefits and barriers or enablers to patient engagement across all levels of involvement, but there was also a “lack of research that reliably investigates whether consumer involvement achieves these intentions and, if so, which methods of consumer involvement are most effective” [45], p. 4. Other studies, however, demonstrated the benefits for patients and decision makers at various levels to have patients engaged in face-to-face discussions and decisions concerning healthcare and health product decisions or issues [42,43]. The sharing of information, experiences and concerns between patients and decision makers was more than educational; it was also informative for healthcare recommendations.

One of the overarching benefits of patient engagement was that it enabled the health system to address the right issues in an appropriate way, design programs, policy and planning activities closely tailored to the needs of both individuals and special populations; achieve better results; and validate outcomes [10,30,43,45,47]. General benefits found in the literature at both an individual and organizational level included better health and treatment outcomes, more appropriate and relevant services, increased legitimacy and credibility of decision making, increased sense of dignity and self-worth, and improved service user satisfaction [47]. It was “assumed that input from consumers in the planning of health care can lead to more accessible and acceptable health services” [45], p. 4.

Many of the benefits and barriers noted in the literature were case or project specific, or derived from stakeholder feedback. For instance, Howe et al. [48] categorized barriers to patient engagement in patient safety initiatives as interpersonal, intrapersonal and cultural. Others categorized barriers as resources (e.g. time and cost), service user or patient issues, organizational issues [42], or system-wide barriers [49]. Both Kovacs Burns [42] and Frankish et al. [50] identified broad barriers to participation in health care decision-making. Many of these challenges or barriers were related to values, assumptions and expectations underlying patient engagement in health authorities, structures and processes associated with decision-making. Kovacs Burns concluded that “Each of these challenges must be anticipated and managed in accordance with the partnership and process aspects that are part of the engagement framework [42]”.

More specific barriers/challenges identified by patients or families, care providers and leaders or administrators, are summarized in Table 4.

**Table 4:** Benefits and barriers to patient engagement for patients, providers, leaders and institutions

**Barriers**	**Benefits**
**Patient barriers**	**Patient benefits**
Personal and professional commitments [42]	Helps improve communications [12]
Patients seen as having the time and resources to participate – not always the case [50]	Better understanding of health services [12]
Health status and self-confidence [10,29]	Commitment to contribute [10]
Time to deal with diagnosis [10]	Patients meet other patients [10]
Financial considerations – need expenses paid [10,42]	Become empowered and valued for expertise and skills [10,42]
Time availability & time for project [10,42]	
Not seeing direct personal benefit [10]	
‘Involvement fatigue’ [10]	
Meeting times (daytime meetings and work) [10,42]	
**Provider barriers**	**Provider benefits**
Negative attitudes toward patient involvement [10,50]	Builds trust and better communication between patients and staff [12]
Lack knowledge of patient involvement [10,31]	Provides information about patient experience to inform planning and service improvement [12]
Dismissive of how patients can contribute and not forthcoming with information/resources [16,50]	Helps to provide accessible and responsive services based on local experience and need [12]
Difficulties/unwillingness to explain complex terminology [16,50]	Enhances patient confidence in health system [12]
Feel threatened by possible reduction of influence, and significant change from medical-model [16,51]	
Difficulties in relinquishing power [32,52]	
Affect on clinician/patient relationship [16]	
**Leader/Instituion barriers**	**Leader/Institution benefits**
Negative attitudes toward patient involvement [51]	More appropriate, better quality and relevant services [9,10,43,45]
Lack of knowledge of how patients may be involved - little training or guidance for professionals in partnership working or joint decision-making [10]	Service responsive to patients’ needs [32]
Tokenism [1,32,50,52]	Policy, research, practice and patient information that includes consumers’ ideas or addresses their concerns [16,45]
Leadership may be questioned either way [42]	Organization is participative, accountable and transparent [16,42]

These were categorized generally into the following areas [47]:

• **Legal -** In general, although a high level of individual patient rights existed regarding healthcare, there was a gap when it came to ‘patient involvement’ as a right.

• **Political -** The lack of or poor political commitment to patient involvement at all levels in the healthcare system and especially at the policy decision level was one of the strongest barriers. Bureaucracy, including administrative procedures, reporting and technical skills required for some engagement activities [43] as well as the “lack of political will and government commitment to ensuring stakeholder engagement in decisions that concern policies or programs” [45], p. 11, were difficult barriers to overcome.

• **Administrative -** Patient involvement was seen as inconvenient and time-consuming interrupting the smooth operation of a hierarchical, bureaucratic organization, especially if there was little or no knowledge about practices of involvement.

• **Professional -** Despite progress towards acceptance of a more important role for patients, attitudes of health professionals remained a strong barrier [29,49]. Negative attitudes might manifest through professionals disengaging, not sharing information or resources, or exerting their power [50]. Much of this negativity could stem from professionals feeling threatened if they had to seek advice from expert patients; that it was a significant change from the medical model they were used to; or that it might question the role of health professionals [16,31,43,48].

• **Communication -** Language, in terms of health literacy and especially with the use of technical terms, was a barrier to patient involvement.

• **Personal -** Characteristics of patients like ethnicity, age, disease and other relevant aspects might lead to discrimination, and therefore lower opportunities for involvement. Other considerations for patient and family involvement included their willingness to partcipate, commitments and time, transportation, wellness and health, language and communication, and fear of health care being jeopardized.

• **Resources -** There were two key aspects: a) the added value of patient involvement had not been quantified in economic terms and, thus had not been adequately compensated and b) meaningful patient involvement required resources.

### Evaluation of patient engagement

Although the literature identified a variety of patient engagement toolkits and guides (Table 2 – A. General Toolkits), the evaluation of them and/or perspectives on those that have been evaluated regarding their use and effectiveness requires further study. An overview of patient engagement evaluation frameworks or algorithms used, evaluation of specific methods, and pilot testing templates for use in community health partnerships were not part of this scoping literature review. Table 2 also contains a list of evaluation toolkits used in various patient or citizen engagement initiatives, most applicable in evaluating the process and outcomes of engagement to make improvements.

Anton et al. [53] examined the development of an assessment framework for public involvement and found that their multi-method study identified a lack of consensus for how public involvement should be evaluated. They believed that evaluation in this sense was contextual and should be tailored to meet the needs of the engagement opportunity and its intended purpose [54]. When evaluating the impact of public involvement policies, Wait and Nolte [55], suggested that it “remains difficult to evaluate, partly due to many policies [being] short lived or very recent. Usually no timeframe or evaluative framework is specified for their assessment” (p. 157). The authors suggested further work was needed to define patient engagement policy objectives and to understand the dynamics of the various stakeholders within the health system in an effort to move healthcare systems closer to those which were responsive to the needs of patients, their families and the public. Cayton [52] suggested that there was “limited evidence to support the argument that patient involvement improves outcomes” and references Coulter and others who have argued that “patient experience, that is, what happened to them, rather than how satisfied they say they are, is a better measure of success” (p. 2). Parsons et al. [56] echoed this sentiment as shown by the challenges they identified in measuring patient engagement in primary care settings.

From the literature, key evaluation components were identified: participation or response rates of consumers, consumer influence on decisions, health care outcomes or resource utilization, consumers’ or professionals’ satisfaction with the involvement process or resulting products, cost, critical factors for success, and limitations of methods or processes. Part of a multistep process, these evaluation components were used to determine whether the engagement opportunity process was effective, the intended goal was achieved, and the engagement outcome had any contextual idiosyncrasies [13,41,57,58]. Rather than evaluation being a step that happens at the end of the engagement opportunity, Sheedy [44] suggested that “Integrating these considerations into the planning process at the outset will save time and frustration at the end, and enable better learning from the process as it is taking place” (p. 33).

### Engagement resourcing

When costing a public dialogue opportunity, the Center for Public Dialogue suggested it was “impossible to provide a ‘standard’ cost estimate” as each engagement opportunity is unique. Considered part of a four-step process, costing for public dialogue opportunities included: consultation planning, testing of materials, implementation, and analysis and evaluation [46], p. 25. Engagement costs might include travel, accommodation, rental space, printing, translation, courier, long distance calls, time of department staff and remuneration of participants.

Considered a challenge to effective service user engagement, McEvoy [43] suggested that adequately resourcing patient engagement enables patients and families (service users) the opportunity to contribute through engagement programs, which if not resourced properly might become tokenistic exercises. The Improvement Leaders’ Guide for Involving Patients and Carers suggested three important considerations: time, financing, and training and support [16].

Sheedy recommended that prior to engaging, all of the components for a successful engagement opportunity should be in place, including time, resources, and capacity. When planning a public or citizen engagement, Sheedy suggested that timing was everything, “while not all citizen engagement projects are time intensive, working with citizens will usually take longer than consulting experts” [44], p. 22. As current budgets did not routinely include funds for engagement, Sheedy also suggested ensuring budget components like transportation, compensating for lost work time, and building internal staff capacity are factored in. All of these aspects were viewed as the necessary ‘infrastructure’ for decreasing barriers for patients to be able to participate and for enhancing their engagement experience.

## Discussion

This systematic scoping review of the published and grey literature provided a glimpse of the complexity of patient/family engagement in health care decision making processes. The literature depicted a large diversity of terms and definitions [8], tools and approaches to patient engagement in different settings, contexts, and purposes (Tables 2 and 3), and barriers and benefits to consider (Table 4). These were captured under seven content themes based on the analysis of selected published and grey literature - ‘*definition of patient engagement’, ‘stakeholder roles and expectations’, meaningful and appropriate engagement’, ‘models of engagement’, ‘benefits and barriers to patient engagement’, ‘evaluation of patient engagement’,* and *‘engagement resourcing’*. These themes could be useful for identifying or naming the content sections of the patient engagement resource kit. In addition, as outcomes of the scoping review the themes identified what knowledge, skills, tools, guides, models and resources existed and could be used or adapted by patients/families, providers or leaders for initiating and developing patient and family engagement. With synthesis of the literature, the sub-themes and information within each of the seven themes were clustered into three main groups more in line with the expected content for a patient engagement resource kit – tools, education and infrastructure.

This scoping literature review provided the opportunity to consider the most appropriate term and *definition of patient engagement* out of 15 choices, to meet the needs of *stakeholders in their roles and expectations*, and to identify different *models of engagement* with tools and guides to adopt or adapt to meet the needs of patient engagement within healthcare organizations such as Alberta Health Services. Over 37 toolkits were identified, of which 14 were general all-purpose patient-related engagement toolkits, and six were patient engagement evaluation toolkits or guides (Table 2). There were also 19 different engagement models, one or more of which could be adapted for use within AHS.Each of the thematic areas within the resource kit also included educational components. For example, key lessons should be heeded from the *benefits and barriers to patient engagement* (Table 4). Creating awareness, clarifying terminology, providing training and tools and guides to understand and become knowledgeable about patient engagement while implementing it, were considered keys to its success. Addressing these lessons with the development of education and awareness tools would help to ensure the success of patient engagement initiatives and strategies. Training and education for all stakeholders, including patients, would help to achieve common language and common grounds for understanding the benefits of and the ‘how-to’ of patient engagement. Providers who have received education would be less threatened and more knowledgeable about how to involve patients and what resources it would take to do so.

Some sources suggested that one of the biggest cultural obstacles to patient engagement was a result of staff and decision-makers making assumptions that patients do not have the knowledge about healthcare operations to be involved in its decision-making processes [48]. “This is a cultural issue that has largely arisen from professionalization and specialization that leads experts to believe that non-experts have nothing or little to contribute” [29], p. 25. Farrell said it best:

Staff education and training will always be integral to any program of change within the [health system]. Initiatives to improve patient and public involvement must address the knowledge, attitudes and skills of professionals and staff at all levels of the service. Hands-on experience could be a powerful way to change attitudes as this opens people’s eyes to the real potential involvement but formal training and education is also crucial [35], p. 39.

If education could increase awareness of the benefits and help to eliminate the barriers to patient engagement, then meaningful and effective patient engagement was more likely to occur. Without *evaluation of patient engagement* initiatives including tools, guides, approaches or process used, and resources to support the process and outcomes, it would be impossible to know what aspects were working well, or which ones needed improvement or changes [16,18]. Obtaining direct patient feedback on the engagement experience was practical for understanding and improving the process and outcomes for patients and others [18].

*Engagement resourcing* of tools and educational or other activities was crucial to engagement success and should be considered when planning any stakeholder engagement. As the engagement of patients and their families was a new area of study, more will continue to be discovered on how best to resource departments invested in patient engagement, the activity itself and the mobilization of the lessons into practice. This was seen as too important to be happening off the side of someone’s desk. Dedicated resources should be applied to ensure the health system had the capacity to be responsive to the needs of patients and their families [49].

The term infrastructure was chosen here for the financial and human resourcing and related supports needed for patient engagement. This term was chosen for several reasons, although the literature does use alternative terms Lansky described the current state of patient engagement ‘infrastructure’ within the United States, and believed that a lack of national health information and an absence of a centralized management and finance system limited the ability for patients to play a more active role [49]. In efforts to build infrastructures which helped to cultivate engagement opportunities, Abelson & Eyles suggested, “building social capital and civic infrastructure is largely a matter of removing the constraints that often truncate that self-organizing process and of improving the space it needs to flourish” [36], p. 19. Like Nova Scotia’s Community Health Boards and Saskatchewan’s Citizen’s Advisory Councils, Alberta legislated the creation of 12 Community Health Councils to provide feedback directly from local Albertans, including patients and their families, about what was working well in the health care system and areas in need of improvement.

Other practical examples of ‘infrastructure’ supporting patient engagement included the emergence of organizational policies, legislation and national health plans, as demonstrated by the Queensland Government endorsement of a Community Engagement Improvement Strategy [23], England’s Health and Social Care Act 2001 which “places a legal duty on health care organizations to make arrangements to involve and consult patients and the public and to develop an ongoing relationship rather than a consultation being a one off” [13], p. 8 and the Scottish Government’s *Better Health, Better Care: Action Plan 2007*[26]. These were examples where infrastructure was supported, and components included the development of patient charters or patient’s rights and responsibilities documents, along with principles and values of patient engagement. The review of the literature would suggest that it was the commitment of organizational leaders which built culture change momentum. Integrated engagement of all stakeholders would help to create learning organizations where patients, families, staff and leaders build collaborative relationships which helped to shift the culture.

## Conclusions

This scoping review on patient engagement, including the identification of key themes and resources relevant to making patient engagement successful, offered a wealth of information that would not only assist in the development of a resource kit for patient engagement but also provide other significant suggestions and recommendations to be considered in the patient engagement process. As follow up to this scoping review and the identification of themes and content (tools, education and infrastructure) items, is the actual development and pilot of the Resource Kit including evaluating the strengths/effectiveness as well as weaknesses of each theme and the content items within each theme. This latter will be an ongoing process within AHS, and the Resource Kit will be remain a dynamic initiative to keep items current and of practical use.

The following were some of the key highlights from this scoping literature review:

• This scoping literature review was comprehensive and unique compared with other literature reviews found on patient engagement. This review included both published and grey literature, and analyzed both in the context of their contributions towards patient engagement in the broadest sense, considering tools, guides, barriers or benefits, and other attributes. Therefore, this scoping review will be filling a gap in the literature concerning patient engagement.

• One of the key findings with the search process was that the term ‘patient engagement’ by itself was inadequate for searching the published and grey literature. Other more commonly used and related terms had to also be part of the search criteria; otherwise, the search would not be adequate, and might not provide access to key sources of patient engagement tools and guides for patients, staff and leaders. This could be interpreted to mean that caution must be taken to not narrow the search terms too quickly but rather be more inclusive as different groups, organizations and researchers have slightly different preferences for ‘patient engagement’ terminology and definitions.

• The selected relevant literature within this scoping review contributed to one or more clustered areas as tools/resources/approaches, education, and support/infrastructure for each of the identified user groups – patients, providers, leaders and AHS Patient Engagement Department. There was a mix of literature relevant across the user groups as well as some specifically targeted literature for specific user groups. This literature will inform the Resource Kit proposed to provide patients, providers and leaders with the information and tools to make patient engagement meaningful and successful.

• Patient engagement is not easy and was in fact quite complex- the literature identified it as being very challenging as it presented with many potential barriers to anticipate or consider in addition to the benefits. From the literature, it was clear what the barriers and benefits were with only some having been evaluated as to their impact. Keeping track of the barriers (legal, political, administrative, professional, communication, personal or resources) as a frame of reference, would help identify tools, guides and strategies to avoid or deal with them before they became a fatal flaw in the patient engagement initiative or process. The goal would be to deal with the barriers and forge a clear path for enablers of patient engagement.

• More research was needed to evaluate the benefits of patient engagement in different settings and contexts. Having an evaluation plan for each patient engagement initiative at the outset would help establish what and how measures were taken and made for both patient engagement processes and outcomes. Evaluation guides and tools were part of this literature review. From an evaluation point of view, Coulter and Ellins conducted a review of the literature for evidence on patient focused quality interventions identifying that while the results of most reviews were positive (beneficial effect), “choosing appropriate criteria to evaluate patient focused interventions is difficult…and the lack of standardized outcome measures hampers comparison of results” [59], p. 24.

• It was recommended that this scoping review and the Resource Kit be dynamic and updated accordingly, especially if it was on the AHS Patient Engagement Department website for patients, providers and leaders to access and implement.

## Competing interests

Katharina Kovacs Burns has no financial relationship with AHS but had academic interests as Principal Investigator for the study. All other authors (Mandy Bellows, Jennifer Gallivan, and Carol Eigenseher), are paid employees or consultants with AHS.

## Authors’ contributions

All authors participated in various capacities within the study as well as with this manuscript. KKB was designated Principal Investigator on the Study, involved with the design, implementation and analysis of all aspects of the study including data gathering, analysis, and resource kit development and piloting. KKB is the lead author on the manuscript and wrote, consulted with co-authors and revised the manuscript for submission. MB was Project Lead for AHS on the study and facilitated all committee meetings, assisted with data collection and analysis, and write up of the scoping literature report. MB was also involved with original discussions on layout of manuscript and with editing and revisions. CE and JG were involved with the review of study results and more involved with making edit and revision suggestions for the manuscript. All were in agreement to the final version of this manuscript being submitted.

## Pre-publication history

The pre-publication history for this paper can be accessed here:

http://www.biomedcentral.com/1472-6963/14/175/prepub

## References

[B1] Luxford K Safron DG Delbanco T |Promoting patient-centered care: a qualitative study of facilitators and barriers in healthcare organizations with a reputation for improving the patient experience Int J Qual Health Care 2011 14 5 510 515 10.1093/intqhc/mzr024 21586433

[B2] Coyle J Williams B Valuing people as individuals: development of an instrument through a survey of person-centredness in secondary care J Adv Nurs 2001 14 3 450 459 10.1046/j.1365-2648.2001.01993.x 11686760

[B3] Alberta Health Services Patient Engagement: A Framework for Engaging Patients with Alberta Health Services 2010 Edmonton, Alberta: Alberta Health Services 1 16

[B4] Arksey H 0’Malley L Scoping studies towards a methodological framework Int J Soc Res Methodol 2005 14 19 32 10.1080/1364557032000119616

[B5] Mays N Roberts E Popay J Fulop N, Allen P, Clarke A, Black N Synthesising research evidence Studying the Organisation and Delivery of Health Services: Research Methods 2001 London and New York: Routledge – Taylor and Francis Group 194

[B6] Creswell J Research Design 2009 3 London: SAGE Publications Inc.

[B7] Guest G Applied thematic Analysis 2012 Thousand Oaks, California: Sage

[B8] Gallivan J Kovacs Burns KA Bellows M Eigenseher C The many faces of patient engagement J Participatory Med 2012 14 e32

[B9] European Patients’ Forum The Value+ Handbook for Project Coordinators, Leaders and Promoter on Meaningful Patient Involvement 2008-2013 European Commission, Directorate General for Health and Consumers, Public Health Programme

[B10] European Patients’ Forum The Value+ Toolkit: for Patient Organisations on Meaningful Patient Involvement Patients Adding Value to Policy, Projects and Services: European Patients’ Forum Online http://www.eu-patient.eu/Documents/Projects/Value+%20Toolkit.pdf

[B11] Oregon Department of Education A Toolkit for Family Involvement in Education Oregon Department of Education Online http://www.ode.state.or.us/opportunities/grants/nclb/fitoolkitpdf.pdf

[B12] Leicester City Primary Care Trust Patient and Public Involvement Toolkit for Staff 2010 Leicester City: National Health Services

[B13] Health & Social Care Regulatory Forum Framework for Public & Service User Involvement in Health and Social Care Regulation in Ireland 2009 Ireland

[B14] Corporate Consultation Secretariat, Health Policy and Communications Branch The Health Policy Toolkit for Public Involvement in Decision Making 2000 Ottawa: Minister of Public Works and Government Services Canada 0-662-29243-X

[B15] Capital Health The Citizen Engagement Progress Measurement Framework Nova Scotia: Capital Health Online 2010 http://www.cdha.nshealth.ca/involving-patients-citizens/our-strategies

[B16] NHS Modernisation Agency Improvement Leaders’ Guide involving Patients and Carers General Improvement Skills 2005 London: Department of Health, NHS

[B17] Gregory J A Framework of Consumer Engagement in Australian Health Policy: Developing a Framework for the AIHPS Study 2006 Australian Institute of Health Policy Studies Research Project Working Paper 2

[B18] Picker Institute Europe Using Patient Feedback: A Practical Guide to Improving Patient Experience U.K: Picker Institute Europe Online http://www.nhssurveys.org/Filestore/documents/QIFull.pdf

[B19] NHS Milton Keynes and Centre for Evidence-based Medicine Public and Patient Engagement Getting it Right 2010 England: Oxford University Available online at: http://www.qualitymk.nhs.uk/patient_public_engagement.htm

[B20] NHS Modernisation Agency Improvement Leaders’ Guide to Measurement for Improvement Online 2002. https://www.fundacionpfizer.org/sites/default/files/pdf/comoevaluarlasmejoras.pdf

[B21] Cambridgeshire Community Services, National Health Service Trust A Staff Guide to Involving Service Users, their Carers and the Public in Cambrideshire Community Services NHS Trust 2010 Available at: http://www.cambscommunityservices.nhs.uk/getting-involved/your-feedback

[B22] Sharpe A Toolkit for Patient Engagement: The International Forum on Quality and Safety in Healthcare Poster at International Forum on Quality and Safety in Healthcare 2002

[B23] Community Engagement Handbook - for Queensland Health District Health Council Members State of Queensland: Queensland Health Online October 2002 http://www.healthissuescentre.org.au/documents/items/2008/04/204586-upload-00001.pdf

[B24] Capital Health Engagement Framework and Toolkit Halifax, Nova Scotia: Capital Health Online 2011. http://www.cdha.nshealth.ca/involving-patients-citizens/documents

[B25] Rotherham Community Health Services Rotherham Community Health Services Handbook for Patient Engagement (Toolkit) 2010 National Health Service

[B26] Scottish Health Council The Participation Toolkit Supporting Patient Focus and Public Involvement in NHS Scotland 2010

[B27] South Central WCC Collaborative PPI Programme Patient and Public Engagement Toolkit for World Class Commissioning NHS Institute for Innovation and Improvement Online http://localdemocracyandhealth.files.wordpress.com/2013/09/ppe-toolit-south-central.pdf

[B28] Leonhardt K Bonin D Pagel P How to Develop A Community-Based Patient Advisory Council 2007 Walworth County: Aurora Health Care

[B29] Forbat L Hubbard G Kearney N Patient and public involvement: models and muddles J Clin Nurs 2009 14 2547 2554 10.1111/j.1365-2702.2008.02519.x 19207798

[B30] Sanna L Assessing the involvement of the patient communicty in European commission co-funded health projects J Ambul Care Manage 2010 14 265 271 10.1097/JAC.0b013e3181e5eb7b 20539154

[B31] While A Real users’ experiences fall short of the mark Br J Community Nurs 2002 14 546 12399707 10.12968/bjcn.2002.7.10.10669

[B32] Happell B Facilitating consumer participation: an approach to finding the ‘right’ consumer Collegian 2010 14 125 130 10.1016/j.colegn.2010.03.001 21046966

[B33] Abelson J Giacomini M Lehoux P Gauvin F-P Bringing ‘the public’ into health technology assessment and coverage policy decisions: from principles to practices Health Poilcy 2007 14 37 50 10.1016/j.healthpol.2006.07.009 16996637

[B34] Chafe R Neville D Rathwell T Deber R Deciding whether to engage the public on health care issues Healthcare Manag FORUM 2008 24 28 10.1016/S0840-4704(10)60271-2 19086482

[B35] Farrell C Patient and Public Involvement in Health: The Evidence for Policy Implementation - A Summary of Results of the Health in Partnership Research Programme 2004 London, England: Department of Health

[B36] Abelson J Eyles J Public Participation and Citizen Governance in the Canadian Health System 2002 Commission on the Future of Health Care in Canada Discussion Paper No. 7

[B37] Culyer A Involving stakeholders in healthcare decisions - the experience of the national institute for health and clinical excellence (NICE) in England and Wales Health Q 2005 14 56 60 10.12927/hcq..17155 16078403

[B38] Institute for Patient- and Family- Centered Care Partnering with Patients and Families to Enhance Safety and Quality – a Mini Toolkit 2010 Maryland: Institute for Patient and Family Centred care Available at: http://www.ipfcc.org/tools/Patient-Safety-Toolkit-04.pdf

[B39] Arnstein Sherry R Ladder of citizen participation J Am Plann Assoc 1969 14 216 224

[B40] International Association for Public Participation IAP2 Spectrum of Public Participation Online 2007 http://c.ymcdn.com/sites/www.iap2.org/resource/resmgr/imported/IAP2%20Spectrum_vertical.pdf

[B41] Rural and Regional Health and Aged Care Services Division Doing it with us not for us - Participation in your Health Service System 2006-09: Victorian Consumers, Carers and the Community Working together with their Health Services and the Department of Human Services 2006 Melbourne, Australia: Rural and Regional Health and Aged Care Services Division, Victorian Government Department of Human Services

[B42] Kovacs Burns K A Canadian Partnership of Patient/Citizen Groups and Government: Engagement in National Health Initiatives and Policy Directions 2006 Edmonton, AB: Faculty of Nursing, University of Alberta

[B43] McEvoy R Keenaghan C Murray A Service User Involvement in the Irish Health Service: A Review of the Evidence 2008 Dublin: Irish Health Service Health Service Executive, [Online] http://www.hse.ie/eng/services/Publications/Your_Service,_Your_Say_Consumer_Affairs/Reports/LiteratureReview.pdf

[B44] Sheedy A Handbook on Citizen Engagement: Beyond Consultation 2008 Ottawa: Canadian Policy Research Networks Inc

[B45] Nilsen E Methods of Consumer Involvement in Developing Healthcare Policy and Research, Clinical Practice Guidelines and Patient Information Material (review) 2010 Ottawa: The Cochrane Collaboration 10.1002/14651858.CD004563.pub2 PMC6464810 16856050

[B46] Centre for Public Dialogue Public Dialogue: A Tool for Citizen Engagement 2000 Washington DC: A Manual for Federal Departments and Agencies

[B47] Vancouver Coastal Health Community Engagement Framework Vancouver: Coastal Health Online http://www.vch.ca/media/CE%20Booklet%202009.pdf

[B48] Howe A Can the patient be on our team? an operational approach to patient involv ment in interprofessional approaches to safe care J Interprof Care 2006 14 527 534 10.1080/13561820600936244 17000478

[B49] Lansky D Patient Engagement and Patient Decision-Making in US Health Care 2003 The Commonwealth Fund and Nuffield Trust Conference

[B50] Frankish James C Kwan B Ratner Pamela A Higgins Joan W Larsen C Challenges of citizen particpation in regional health authorities Soc Sci Med 2002 14 1471 1480 10.1016/S0277-9536(01)00135-6 12061482

[B51] Abelson J Forest PG Eyles J Smith P Martin E Gauvin FP Deliberations about deliberative methods: issues in the design and evaluation of public participation processes Soc Sci Med 2003 14 239 251 10.1016/S0277-9536(02)00343-X 12765705

[B52] Cayton H Patient Engagement and Patient Decision-Making in England 2004 Department of Health for England, The Commonwealth Fund/Nuffield Trust

[B53] Anton S McKee L Harrison S Farrar S Involving the public in NHS service planning J Health Organ Manag 2007 14 470 483 10.1108/14777260710778989 17933377

[B54] Venuta R Graham Ian D Involving citizens and patients in health research J Ambul Care Manage 2010 14 215 222 10.1097/JAC.0b013e3181e62bd7 20539148

[B55] Wait S Nolte E Public involvement policies in health: exploring their conceptual basis Health Econ Policy Law 2006 14 149 162 10.1017/S174413310500112X 18634687

[B56] Parsons S Winterbottom A Cross P Redding D The Quality of Patient Engagement and Involvement in Primary Care 2010 London: The King’s Fund

[B57] Phillips S Orsini M Mapping the Links: Citizen Involvement in Policy Processes CPRN discussion paper no. F|21 2002 Ottawa, ON: Canadian Policy Research Networks Inc

[B58] Health WA Consumer Carer and Community Engagement Framework: for Health Services, Hosptials and WA Health Following Consultation across WA health Government of Western Australia, Department of Health Online April 2007 http://www.health.wa.gov.au/hrit/docs/publications/WA_Health_Consumer_Apr07.pdf

[B59] Coulter A Ellins J Effectiveness of strategies for informing, educating and involving patients Br Med J 2007 14 24 27 10.1136/bmj.39246.581169.80 17615222 PMC1910640

